# Mapping as a tool for predicting the risk of anthrax outbreaks in Northern Region of Ghana

**DOI:** 10.11604/pamj.supp.2016.25.1.6205

**Published:** 2016-10-01

**Authors:** Ayamdooh Evans Nsoh, Ernest Kenu, Eric Kofi Forson, Edwin Afari, Samuel Sackey, Kofi Mensah Nyarko, Nathaniel Yebuah

**Affiliations:** 1 Veterinary Services Directorate Tamale, P.O. Box 241, Tamale, Northern Region, Ghana; 2 Ghana Field Epidemiology and Laboratory Training Programme, Tamale, Northern Region, Ghana; 3 University of Ghana, College of Health Sciences, School of Public Health, Accra, Ghana; 4 Rudan Engineering Limited, 156 Atomic-Haatso Road, P.O. Box CT 828, Cantonments, Accra, Ghana; 5 University of Ghana, School of Public Health, Accra, Ghana

**Keywords:** Anthrax, spatial risk mapping, Northern Region, soil pH, temperature, rainfall, outbreak, surveillance, B. anthracis

## Abstract

**Introduction:**

Anthrax is a febrile soil-born infectious disease that can affect all warm-blooded animals including man. Outbreaks of anthrax have been reported in northern region of Ghana but no concerted effort has been made to implement risk-based surveillance systems to document outbreaks so as to implement policies to address the disease. We generated predictive maps using soil pH, temperature and rainfall as predictor variables to identify hotspot areas for the outbreaks.

**Methods:**

A 10-year secondary data records on soil pH, temperature and rainfall were used to create climate-based risk maps using ArcGIS 10.2. The monthly mean values of rainfall and temperature for ten years were calculated and anthrax related evidence based constant raster values were created as weights for the three factors. All maps were generated using the Kriging interpolation method.

**Results:**

There were 43 confirmed outbreaks. The deaths involved were 131 cattle, 44 sheep, 15 goats, 562 pigs with 6 human deaths and 22 developed cutaneous anthrax. We found three strata of well delineated distribution pattern indicating levels of risk due to suitability of area for anthrax spore survival. The likelihood of outbreaks occurrence and reoccurrence was higher in Strata I, Strata II and strata III respectively in descending order, due to the suitability of soil pH, temperature and rainfall for the survival and dispersal of B. anthracis spore.

**Conclusion:**

The eastern corridor of Northern region is a Hots spot area. Policy makers can develop risk based surveillance system and focus on this area to mitigate anthrax outbreaks and reoccurrence.

## Introduction

Anthrax is a soil-borne infectious disease that can present as peracute, acute, subacute or chronic febrile illness of all warm blooded animals including man. Bacillus anthracis, the causative agent of anthrax is a multihost pathogen affecting human, livestock and wildlife populations. Anthrax remains endemic in many African countries causing significant losses in domestic animal populations [1]. In Africa it has been reported in Cameroon [2], the Mago National Park Omo in Ethiopia [3], in Tanzania [4] and at the Selousa National Reserve in Tanzania [5], the Luangwa valley Zambia [6], the Kruger National Park in South Africa [7], Etosha National Park in Namibia [8]. West and Central Africa [9] have reported a new Bacillus anthracis found in wild chimpanzees and a gorilla. Anthrax outbreaks in Ghana have been reported since 1988 in the World Anthrax Data Site and impacts negatively on the economy of the livestock industry and public health. Anthrax is considered a major non-contagious, zoonotic disease since ancient times. Outbreaks in Northern Ghana have caused devastating economic effects in the livestock sector due to ban on livestock movement and slaughter and caused alarming public health concern [10]. Livestock production in northern Ghana is vital in providing food security and economic development to the people. The region holds 75% of the nation’s cattle, 54% sheep, 57% goats, 55% pigs. An estimated 89% of the farmers in Nnorthern Ghana rear livestock as well crops [11]. Over a third of the income of farm families is derived from livestock production [12]. However, livestock development in this area is challenged by diseases and high mortality. Northern region has been identified as an area with frequent Anthrax outbreaks in livestock [13] which results in high mortality, production and reproduction losses. The ecology and outbreaks pattern of Anthrax is not well known in Northern Ghana. In different parts of the world, anthrax cases have been directly associated with sudden rainfall and soil nutrient availability [14] as well as temperature [15]. These variables have been incorporated into GIS tools to map the suitability of the environment for B. anthracis spore survival, predict risk of outbreaks and identify hotspots in studies in regions such as Kazakhstan [16] and Saskatchewan [17]. Knowledge on the hotspots of anthrax within northern Ghana will help predict anthrax outbreaks leading to improvement in livestock production. The main objective of this work was to use routinely generated climatic data to build predictive risk maps to identify anthrax hotspot areas in Northern Region of Ghana.

## Methods

**Study design:** we carried out a descriptive cross-sectional study which involved 10 year record review of rainfall, temperature from Meteorological service department and soil pH records from Soil Research institute covering the Northern part of Ghana. Anthrax outbreaks records in the Region from January 1, 2003 to December 31, 2012 were reviewed from district Veterinary offices.

**Study sites:** Northern Region of Ghana lies between longitude 1° 12” E and 3° 15” W and latitude 10° 30” N and 11° 10” N. The region has a single rainy season that begins in May and ends in October. The soil types are savannah Ochrosols. We used well trained and experienced field workers for the extraction of records on temperature and rainfall for the 10 year period to ensure quality. Two independent Meteorological officers cross checked rainfall and temperature records with the original files and all the necessary corrections were made. All Anthrax outbreaks coordinates which were not well recorded were retaken with the E-trex GARMIN Geographical Positioning System receiver.

### Data analysis

We used Microsoft Excel for editing, validation, verification and descriptive data analysis to summarize the data. The monthly mean values of rainfall, temperature and soil pH were calculated per district for the study period. The descriptive summary of the outbreaks was done by pooling the respective monthly outbreaks data over the period under study to determine monthly occurrences. For seasonal analysis, the year was divided into Rainy and Dry seasons. For yearly trend analysis, the outbreaks of the respective years were added together. The district outbreak frequency was calculated as the number of outbreaks per district during the 10 years period. The geo-referenced data of outbreaks sites were used to generate distribution map of the spatial spread of the outbreaks using Arc GIS 10. We queried an existing shape file indicating the boundary of Ghana using ArcGIS software to carve out the boundary of the northern region. The mean values in excel were joined to their respective spatial district boundary with the help of the “join” tool in ArcGIS. The mean value of each district was concentrated in the centroid of the district. These values were interpolated using the kriging method in ArcGIS [18] to get values within the entire region. With the help of the reclassify tool in ArcGIS, each factor rainfall, temperature and soil pH was categorized into three classes (1, 2 and 3). Constant raster of values 0.5, 0.3 and 0.2 were created as weights for soil pH, rainfall and temperature respectively. The re-classified layers of soil pH, rainfall and temperature were multiplied by their respective constant raster value using the spatial analyst tool in Arc GIS. The product of the soil pH and its standard weight was added to the product of the rainfall and its standard weight. The sum of the two layers produced the Soil pH and Rainfall predictive map. The Soil pH and Rainfall predictive map was finally added to the product of the temperature and its standard weight to get the final predictive map. The goodness of fit of the model was assessed by overlaying the predictive map with the spatial distribution of Anthrax outbreaks in the study area. The final predictive map (Fig 3B) was stratified for anthrax outbreaks surveillance considering suitability of environment for anthrax spore survival. Based on this, the region was divided into High risk areas as hot spots (stratum I), moderate risk areas as stratum II and the low risk area as stratum III.

## Results

In this study, within the 10-year period, there were 43 confirmed outbreaks. There were deaths of 131 cattle in 26 outbreaks, 44 sheep in 12 outbreaks, 15 goats in 4 outbreaks, 562 pigs in 2 outbreaks and 6 human deaths in 3 outbreaks. Twenty two people developed cutaneous anthrax (Table 1).

**Table 1:** 2003-2012 climatic and Anthrax outbreaks characteristics in Northern Region, Ghana

Year	Mean Rainfall (mm)	Mean Temperature (^°^C)	outbreaks	Cattle	sheep	goats	pigs	Human deaths
**2003**	1088.61	27.76	0	0	0	0	0	0
**2004**	1194.00	28.14	0	0	0	0	0	0
**2005**	935.02	30.49	7	38	1	0	0	1
**2006**	923.13	28.56	5	17	16	0	0	1
**2007**	1034.92	28.31	5	15	9	3	0	3*
**2008**	1215.98	28.16	6	20	6	11	0	1
**2009**	1160.15	28.21	2	2	1	1	0	0
**2010**	1304.41	28.23	5	6	2	0	0	0
**2011**	975.94	28.41	9	21	2	0	3	0
**2012**	1119.56	28.12	4	12	7	0	559	0
**Total**	1095.17*	28.44*	43	131	44	15	562	6

* 22 human cutaneous anthrax cases occurred

**Figure 1:**
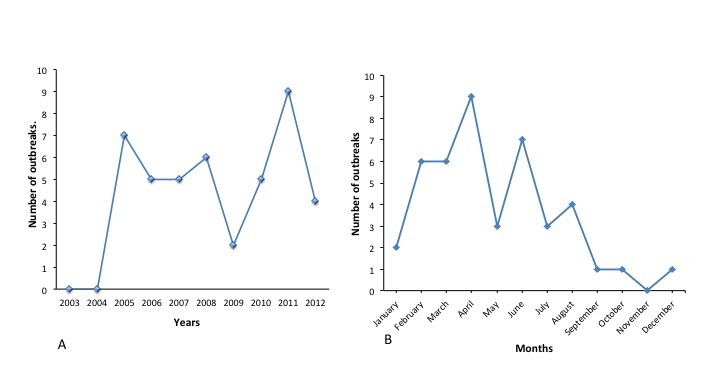
Anthrax outbreaks in Northern Region of Ghana by years **(A)** and months **(B)**, 2003 -2012

The highest number of outbreaks occurred in 2011 with the least in 2009. There were no outbreaks in 2004 and 2003 (Fig 1A). The highest number of outbreaks occurred in April and the lowest in November (Fig 1B).

**Figure 2:**
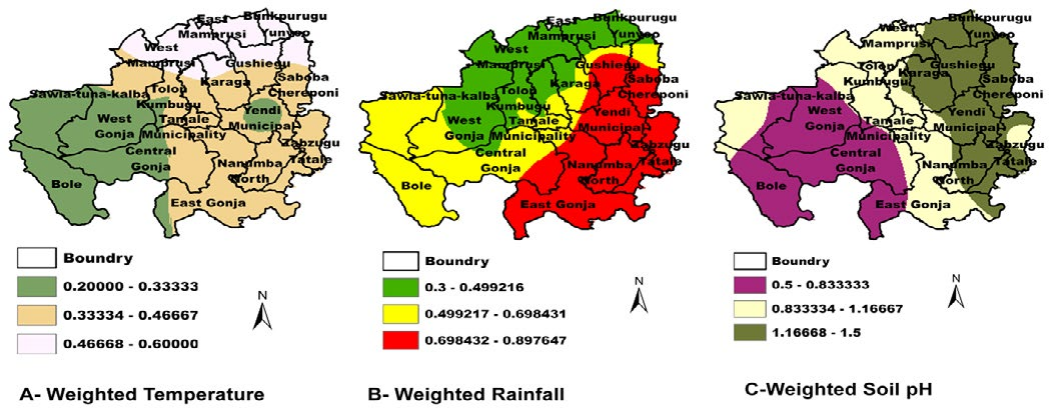
Rainfall **(A)**, temperature **(B)** and soil pH **(C)** distribution in Northern Region, Ghana, 2003-2012

**Figure 3:**
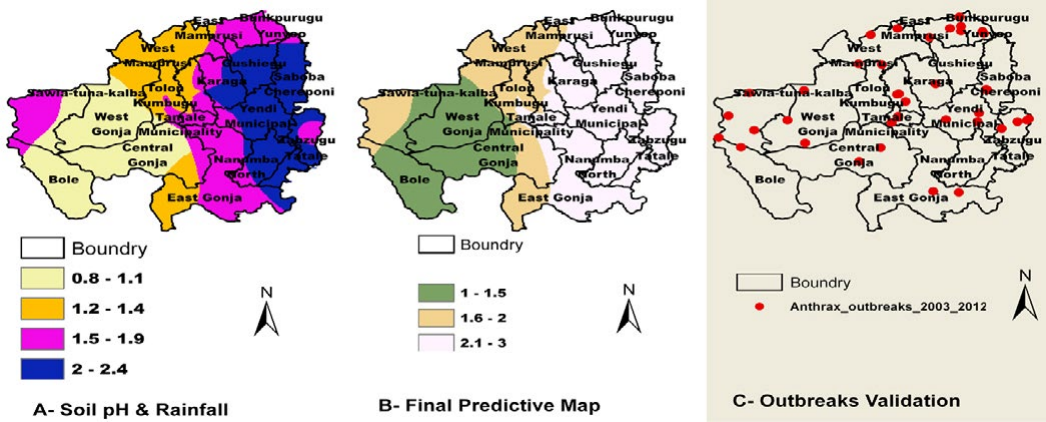
Soil pH and rainfall predictive **(A)**, final predictive map of anthrax outbreaks **(B)** and goodness of fit map of Anthrax outbreaks distribution

Bunkpurugu-yunyoo, Savelugu-Nanton and Yendi experienced the highest number of outbreaks 5 (11.6%), Six out of twenty districts did not experience outbreaks during this period; Saboba-Chereponi, Karaga, Nanumba north and south and Tolon-Kumbungu. Some districts such as East Gonja, Saboba, Yendi, Nanumba North and South experienced very high rainfall and might have experienced flooding and erosions. Eight out of twenty districts; Bole, Sawla-Tuna-Kalba, Tolon-Kumbungu, West Gonja, Central Gonja, Tamale municipal, Savelugu-Nanton and Karaga experienced medium monthly mean rainfall. The rest of the 3 districts, East and West Mamprusi and Bunkpurugu-yunyoo experienced low rainfall (Fig 2B). Bole, Sawla-Tuna-Kalba, West and Central Gonja experienced low temperatures but Tolon-Kumbungu, Tamale municipal, East Gonja, Nanumba North and South, Zabzugu-Tatale, Saboba-Chereponi experienced medium temperatures of 27.9°C – 28.6°C. The other districts Gushiegu, Karaga and Savelugu-Nanton experienced high temperatures ranging from 28.7°C - 29.5°C and very high temperatures occurred in East and West Mamprusi to Bunkpurugu-yunyoo recording temperatures between 29.6°C-30.7°C. (Fig 2A). Areas with low soil pH covers Bole, Central and West Gonja and the Southern part of East Gonja precisely Kpandai. The medium soil pH covers East Gonja, Tamale Municipal, Tolon-Kumbungu, and Sawla-Tuna-Kalba. The areas classified as high soil pH are West Mamprusi, East Mamprusi, Savelugu-Nanton, Yendi and Zabzugu-Tatale. The districts ranked very high soil pH are Bunkpurugu-yunyoo, Gushiegu, Karaga, and Saboba-Chereponi. (Fig 2C) Based on the Kriged generated soil pH+Rainfall predictive map (Fig 3A) the region was divided into 4 classes by natural breaks and the final predictive map was divided into three stratums by risk: Stratum I (High risk area): East Mamprusi, Bunkpurugu-yunyoo, Gushiegu, Karaga, Yendi, Saboba-Chereponi, Tamale Municipal, East Gonja, Nanumba North and South and Zabzugu-Tatale. Stratum II (Medium Risk): West Mamprusi, Tolon-Kumbungu, Savelugu-Nanton, Kpandai and Sawla-Tuna-Kalba. Stratum III (Low Risk): West Gonja, Central Gonja and Bole. We generated validation map (Fig 3C) made up of the 10 years Anthrax outbreaks distribution map in the Northern Region.

## Discussion

The identification of hotspot areas is important for the control and eradication of anthrax. This study therefore clearly provides an anthrax spore suitability mapping and hotspot identification in an endemic area in Ghana. In total 43 outbreaks were recorded during the 10 years period of study with a peak in April and the highest in Bunkpurugu-yunyoo, Savelugu-Nanton, and Yendi and the highest outbreaks occurred in 2011. Our findings show a significance influence of seasonal variation on anthrax outbreak occurrences consistent with Chikerema [19]. The majority of the outbreaks occur at end of the dry season and early part of the rainy season which is associated with perennial shortage of livestock feed forcing animals to graze very low and animals are more likely to acquire the Anthrax spores. The records in a single outbreak, shows high pig mortality of 500 in Bole. This could be attributed to poor biosecurity measures which includes pigs being reared on free range and can easily unearth shallow buried anthrax carcasses [4]. Bole and Sawla-Tuna-Kalba are on the main route from Upper West where animals are being sold and transported to the southern sector. Similar studies carried out elsewhere [20] utilized multiple environmental variables including measures of temperature, precipitation, soil, and vegetation to establish a potential distribution model of B. anthracis in the United States based on the relationship between known occurrence data and environmental variables in proximity to the data. Outbreaks have been associated with heavy rains and flooding which are hypothesized to unearth spores [21]. East Mamprusi, Bunkpurugu-yunyoo, Gushiegu, Karaga, Yendi, Saboba-Chereponi were the hardest affected area during the August, 2007 floods in the country and that area remains prone to flood [22] The map produced (Fig 2C) illustrated that with the soil pH districts such as East Mamprusi, Bunkpurugu-yunyoo, Gushiegu, Karaga, Yendi, Saboba-Chereponi were more suitable for the anthrax spore survival. Our soil pH finding is consistent with other works, that soils with pH above 6.1 to alkaline have been shown to be important geographical determinants of anthrax occurrence because of increased spore survival [15, 23]. Some work has found Seroprevalences in dogs consistently showing circulation of anthrax in areas where no human or livestock anthrax cases were reported but with high soil alkalinity [4]. Although the results of rainfall as a predictor seems a poor explanatory variable in other research work [24], contrary, our results established that the reoccurrence and outbreaks of Anthrax and rainfall have a well establish association. The soil pH and rainfall predictive map generated was stratified into three classes similar to [25]. Similar results has been documented in Canada [15] that anthrax outbreaks may be associated with alkaline soil pH and high moisture due to rainfall. Similar to work in Zimbabwe [19] we divided the region into high risk area (Stratum I) Medium risk area (Stratum II) and Low risk area (stratum III). Notably in stratum II are three districts that has not experienced any outbreak but once occurred the spore has the potential to survive for longer period due to the bioclimatic and the soil pH suitability for its survival.

## Conclusion

In the light of these results, the kriged map has identified the following districts: East Mamprusi, Bunkpurugu-yunyoo, Gushiegu, Karaga, Yendi, Saboba-Chereponi, Tamale Municipal, East Gonja, Nanumba North and South and Zabzugu-Tatale as hot spot areas. The risk of Anthrax outbreak and reoccurrence is much higher than the rest of the districts.
